# Spinal Actions of Lipoxin A_4_ and 17(R)-Resolvin D1 Attenuate Inflammation-Induced Mechanical Hypersensitivity and Spinal TNF Release

**DOI:** 10.1371/journal.pone.0075543

**Published:** 2013-09-24

**Authors:** Sally Abdelmoaty, Gustaf Wigerblad, Duygu B. Bas, Simone Codeluppi, Teresa Fernandez-Zafra, El-Sayed El-Awady, Yasser Moustafa, Alaa El-din S. Abdelhamid, Ernst Brodin, Camilla I. Svensson

**Affiliations:** 1 Department of Physiology and Pharmacology, Karolinska Institutet, Stockholm, Sweden; 2 Department of Molecular Biochemistry and Biophysics, Karolinska Institutet, Stockholm, Sweden; 3 Department of Pharmacology and Toxicology, Faculty of Pharmacy, Suez Canal University, Ismailia, Egypt; 4 Department of Clinical Pathology, Faculty of Medicine, Suez Canal University, Ismailia, Egypt; University of Kentucky Medical Center, United States of America

## Abstract

Lipoxins and resolvins have anti-inflammatory and pro-resolving actions and accumulating evidence indicates that these lipid mediators also attenuate pain-like behavior in a number of experimental models of inflammation and tissue injury-induced pain. The present study was undertaken to assess if spinal administration of lipoxin A_4_ (LXA4) or 17 (R)-resolvin D1 (17(R)-RvD1) attenuates mechanical hypersensitivity in the carrageenan model of peripheral inflammation in the rat. Given the emerging role of spinal cytokines in the generation and maintenance of inflammatory pain we measured cytokine levels in the cerebrospinal fluid (CSF) after LXA4 or 17(R)-RvD1 administration, and the ability of these lipid metabolites to prevent stimuli-induced release of cytokines from cultured primary spinal astrocytes. We found that intrathecal bolus injection of LXA4 and17(R)-RvD1 attenuated inflammation-induced mechanical hypersensitivity without reducing the local inflammation. Furthermore, both LXA4 and 17(R)-RvD1 reduced carrageenan-induced tumor necrosis factor (TNF) release in the CSF, while only 17(R)-RvD1attenuated LPS and IFN-γ-induced TNF release in astrocyte cell culture. In conclusion, this study demonstrates that lipoxins and resolvins potently suppress inflammation-induced mechanical hypersensitivity, possibly by attenuating cytokine release from spinal astrocytes. The inhibitory effect of lipoxins and resolvins on spinal nociceptive processing puts them in an intriguing position in the search for novel pain therapeutics.

## Introduction

Inflammatory mediators released subsequent to nerve and tissue injury are instrumental for the induction, enhancement and propagation of pain. However, recent work shows that it is not only the inflammatory, but also the anti-inflammatory factors that determine the degree of pain and the propensity of pain chronification [1-6]. New families of endogenous anti-inflammatory lipid mediators, including lipoxins and resolvins, have been identified during the recovery phase of inflammation. Lipoxins are derived from the ω-6 poly-unsaturated fatty acid (PUFA), arachidonic acid, whereas, resolvins are classified into the D or E series and are derived from the ω-3-PUFAs docosahexaenoic acid (DHA) and eicosapentaenoic acid (EPA), respectively. In the presence of aspirin, ω-3 and ω-6-PUFAs are converted to the aspirin triggered form of lipoxins and resolvins through a cyclooxygenase-2 dependent pathway. Aspirin triggered lipoxins and resolvins, also denoted as the R-form, are more resistant to dehydrogenation and thereby more stable than the native forms [7].

Lipoxins and resolvins have anti-inflammatory and pro-resolving actions in animal models of inflammatory diseases such as colitis, periodontitis and asthma through their ability to reduce the recruitment of neutrophils and eosinophils, and stimulate monocytes and macrophages to perform phagocytosis of microorganisms and apoptotic cells without releasing pro-inflammatory mediators (reviewed in 8). Accumulating reports show that lipoxins and resolvins are not only coupled to the resolution of inflammation but also play important roles in the modulation of experimentally-induced inflammatory pain. When lipoxins and resolvins are administrated systemically or locally, they reduce carrageenan-induced heat hypersensitivity [2,9,10] and complete Freund’s adjuvant (CFA)-induced mechanical hypersensitivity [11] in rats. In addition, a reduction in edema was observed after the systemic administration of lipoxins [2] and local administration of resolvin E1 (RvE1) [4] suggesting the anti-nociceptive properties could be coupled to the local anti-inflammatory effects of these lipid mediators. However, spinal (intrathecal, i.t.) injection of both lipoxins and resolvins attenuate pain-like behavior without reducing the peripheral inflammation [2,4], which indicates that they can alter pain signaling through spinal mechanisms. For example, i.t. injection of lipoxin A_4_ (LXA4) or aspirin-triggered LXA4 reduces carrageenan-induced thermal hyperalgesia [2], neuropathic pain-associated mechanical and thermal hypersensitivity following dorsal root ganglia (DRG) compression [12] and bone cancer pain-associated mechanical hypersensitivity [13] in rats. Further, spinal injection of resolvin D1 (RvD1), RvD2 and RvE1 reduced formalin-induced flinching, capsaicin-induced nocifensive behavior and CFA-induced thermal and mechanical hypersensitivity in mice [4,9,14] and post-operative tactile hypersensitivity and hyperalgesia in rats [15].

Lipoxins and resolvins are ligands for several receptors and thus the exact mechanisms of lipoxin and resolvin-induced inhibition of inflammatory pain warrants careful investigation. Soon after the discovery of LXA4 and RvD1, these lipid metabolites were found to exert their actions via the G protein-coupled receptor, formyl peptide receptor 2/Lipoxin A4 receptor (FPR2/ALX) and the G-protein coupled receptor 32 (GPR32) [16-18]. FPR2/ALX is also known as the human formyl-peptide receptor like-1 (FPRL-1) and ALXR. Both mRNA and protein levels of FPR2/ALX have been detected in rat primary astrocytes [19,20] and localized to astrocytes in vivo by immunohistochemistry [2,13]. Less is known about GPR32, which has been identified as a receptor for LXA4 and 17 (R)-RvD1 in humans but not yet isolated in rodents. In addition to acting on FPR2/ALX and GPR32, recent data points to the anti-nociceptive actions of resolvins also being mediated through several different transient receptor potential (TRP) channels. RvE1 and RvD1 suppress TRPA1, TRPV1 and TRPV4 mediated activity in primary afferents [4,9,14] and 17(R)-RvD1 suppresses TRPV3 mediated activity in keratinocytes [10].

There is mounting evidence that astrocytes play an important role in pain processing. Spinal astrocytes show signs of activation in response to peripheral painful insults and they have the capacity to produce various mediators, including cytokines and chemokines, which contribute to injury-induced hypersensitivity [21]. Cytokines and chemokines regulate nociceptive mechanisms both in the peripheral and central nervous system, and for example, blockage of spinal TNF-α and IL-1β actions attenuates persistent pain behavior in a number of different experimental models of pain (reviewed in 22). These cytokines have been suggested to serve as “gliotransmitters” in the cross-talk between glia and neurons in experimental models of pain [23]. Previous studies show that lipoxins and resolvins reduce cytokine and chemokine production in experimental models of inflammatory conditions as well as in pain models. For example, it has been demonstrated that i.t. administration of lipoxins reduces elevation of cytokine mRNA and protein in the spinal cord and DRG in models of nerve injury and bone cancer- induced pain [12,13]. Additionally, resolvins attenuate cytokine and chemokine synthesis at the site of inflammation when administrated systemically and in the spinal cord when delivered intrathecally in models of inflammation and pancreatitis-induced pain, respectively [11,24]. Thus, resolvins and lipoxins could potentially prevent or reverse spinal sensitization, at least partially, by interfering with cytokine and chemokine production.

From an intracellular perspective, signaling through the NF-κB and mitogen activated protein kinase (MAPK) pathways is altered in the presence of lipoxins and resolvins [25-30]. These pathways are activated in the spinal cord subsequent to peripheral inflammation and have been linked to spinal cytokine and chemokine production [31]. Inhibition of spinal NF-κB and the MAPKs (p38, ERK and JNK) attenuates inflammation-induced pain behavior [31,32]. Hence, spinal release of cytokines and chemokines provides a potential link between NF-κB and MAPK activation and spinal sensitization that could potentially be regulated by resolvins and lipoxins.

We have previously demonstrated that i.t. injection of lipoxins attenuates carrageenan-induced thermal hypersensitivity [2]. Our current work examines if spinal administration of LXA4 and 17(R)-RvD1, a more stable analogue of RvD1, can attenuate carrageenan-induced mechanical hypersensitivity and release of cytokines in the CSF in rats. As pain behavior in the carrageenan model is resolved within 24 hours we examined if spinal FPR2/ALX expression changes over time subsequent to carrageenan injection, as alteration in the expression of this receptor may be important for the capacity of LXA4 and 17(R)-RvD1 to dampen the pain process. Lastly, given that FPR2/ALX is expressed in astrocytes, we examined if LXA4 or 17(R)-RvD1 attenuated stimulus-induced TNF release in rat primary astrocyte cell cultures.

## Materials and Methods

### Animals

Adult male Sprague Dawley rats, weighing 250-300 g, were purchased from Charles River, Germany and Indianapolis, USA. The rats were housed in groups (3-5 per cage) and maintained on a 12-h light/dark cycle with free access to food and water. All experiments were performed during the light cycle.

### Ethics Statement

All experiments were carried out according to protocols approved by the local Ethical Committee for animal experiments (Stockholms Norra Djurförsöksetiska Nämnd, ethical permit number: N4/09) and the Institutional Animal Care and Use Committee of the University of California, San Diego (ethical permit number: S02075R) under the Guide for Care and Use of Laboratory Animals, National Institutes of Health publication 85-23, Bethesda, MD, USA. All efforts were undertaken to minimize the suffering of the experimental animals, and proper enrichment of the environment was introduced.

### Carrageenan-induced inflammation

To induce a state of local inflammation, 100 µl of a 2% carrageenan solution (Lambda, Wako chemicals, Osaka, Japan; w/v in physiological saline) was injected subcutaneously into the dorsal aspect of the left hind paw under brief isoflurane anesthesia (2–4%).

### Intrathecal injections

Intrathecal injections were performed under isoflurane anesthesia (2-4%) by introducing a 28-gauge needle connected to a 50 µl Hamilton syringe into the space between the L4 and L5 vertebra and slowly injecting 10 µl of LXA4, 17(R)-RvD1, or vehicle (phosphate-buffered saline; PBS). Drugs were administered intrathecally 10 minutes before carrageenan injection to the hind paw. LXA4 and 17(R)-RvD1 (Cayman Chemicals, Michigan, USA) were prepared according to the provider’s instructions. Briefly, less than one hour before i.t. injection, 17(R)-RvD1 and LXA4, supplied as 0.1 µg/µl in ethanol, were dried under nitrogen gas stream and dissolved in PBS (pH 7.2) at concentrations ranging from 0.1-1 µg/µl for LXA4 and 0.3–300 ng/µl for 17(R)-RvD1. All drugs were injected in a volume of 10 µl, followed by 10 µl saline to flush the catheter.

### Assessment of mechanical hypersensitivity and paw inflammation

For measurement of tactile thresholds, rats were placed in individual Plexiglas compartments (26 × 11 × 20 cm) with wire mesh bottoms. Animals were habituated to the testing conditions for 60 minutes daily for at least two days before the experiment started. Animals were allowed a 30 minutes acclimatization period in the test cages prior to assessment of baseline thresholds. Thresholds were assessed on two consecutive days before testing in order to habituate the rats to the testing procedure. Baseline measurements for the experiment were performed 30 minutes prior to carrageenan injection and used as the 0 hour value in the graphs. Von Frey filaments were used for the assessment of mechanical hypersensitivity according to the Dixon up-down method [33]. Briefly, 8 optic glass fiber filaments (Marstock OptiHair®, Schriesheim, Germany) with buckling forces between 0.5 and 16 g were applied perpendicularly to the mid-paw plantar surface (L4 dermatome) until the filament was slightly bent and held there for 4-6 s. Testing was started with the 2.0 g hair and the next filament was applied when the animal was calm with both hind paws placed on the grid. A positive response was noted when the rat displayed a brisk withdrawal from the stimulus. The experimenter was blinded to drug treatments during all behavioral testing. The 50% probability withdrawal threshold (force of the von Frey hair to which an animal reacts to 50% of the presentations) was determined and plotted versus time.

The data was also expressed as the hyperalgesic index for the time period 0-6 hours. The hyperalgesic index represents the area under the time effect curve after stimulation in which the “percent reduction from baseline response threshold” is plotted against time. The resulting metric is percentage change x hour. The formula for calculating the percentage change is (baseline threshold − post-drug threshold) × 100 / baseline threshold, where threshold is expressed in grams. Increasing values reflect increasing hyperalgesia. Local inflammation was assessed by measurement of the vertical thickness at the metatarsal level of the hind paw (in millimeters) using vernier calipers.

### CSF collection and cytokine analysis

Under Isoflurane anesthesia rats were placed in the prone position and the spinous process at L1/L2 was identified as a tactile landmark. A midline skin incision, approximately 3 cm in length, was made caudally from the landmark to expose the interspinous space at L4/L5. The L4/L5 interspinous ligament and the L5 spinous process were carefully removed. While elevating the L4 spinous process with forceps to widen the L4/L5 interlaminar space, the tip of a pulled capillary tube was obliquely introduced into the i.t. space. The jugular veins were compressed to increase the i.t. pressure and 40-50 µl of clear CSF was collected by capillary action. The CSF was transferred to Eppendorf tubes and immediately frozen on dry ice and stored at -80 °C. Rat interleukin (IL)-1β, IL-4, IL-8, IL-13, Interferon-γ (IFN-γ) and tumor necrosis factor (TNF) were measured using an electrochemiluminescence assay (multiplex platform cat. No. K15014C, Mesoscale Discovery (MSD) Gaithersburg, MD, USA) and outputs were measured with a Sector Imager 2400 apparatus (MSD) according to the manufacturer’s instructions. TNF in cell culture media was measured using a single-plex platform (cat. No. K153BHC-1, MSD) and the protocol modified in order to increase sensitivity (lower detection limit 2 pg/ml).

### Astrocyte primary cell cultures

Human (ScienCell Research Laboratories, Carlsbad, CA, USA) and rat primary astrocytes were used for cell culture experiments. Rat astrocytes were isolated from spinal cords using the previously described protocol [34]. Briefly, after ejection of spinal cords from the vertebral column using a saline-filled syringe, meninges were removed and the spinal cords transferred to complete astrocyte medium (AM) consisting of basal AM supplemented with 2% FBS, 50 U/ml penicillin, 50 µg/ml streptomycin and astrocyte growth supplement that provided a final concentration of 10 µg/ml of BSA, 10 µg/ml of apo-transferrin, 5 µg/ml of insulin, 2 ng/ml FGF-2, 2 ng/ml IGF-I, 1 µg/ml hydrocortisone and 20 nM progesterone (ScienCell Research Laboratories). The spinal cords were cut into small pieces and the cells dissociated with 0.25% trypsin-EDTA (Sigma, St. Louis, MO, USA) at 37°C for 10 minutes followed by mechanical trituration. The cells were cultured in complete AM for 11 days and, on days 12 and 13, cells were shaken at 450 r.p.m. for 6h and complete AM was replaced to remove microglia. On day 14 the cells were trypsinized, replated into 24 well-plates and maintained in complete AM until 70-80% confluency was reached. The cells were subjected to 24 hours serum starvation (DMEM supplemented with 50 U/ml penicillin, 50 µg/ml streptomycin and 1mM sodium pyruvate [Invitrogen]) prior to RNA isolation.

For TNF analysis, primary rat astrocytes were serum starved for 48 hours and LXA4 (500 nM) or 17(R)-RvD1 (500 nM) were added 30 minutes prior to cell stimulation with IFN-γ (Sigma I-3275; 1000 U/ml) lipopolysaccharide (LPS, Chondrex, Redmond, WA, USA; 2 µg/ml), or IFN-γ (1000 U/ml) added 4 hours prior to LPS (2 µg/ml) stimulation. Cell culture media was collected 24 hours later, centrifuged at 14000 rpm at 4 °C for 10 minutes and the cell free supernatant stored at -80 °C until analysis. For MAPK analysis, human astrocytes were serum starved for 24 hours and 17(R)-RvD1 (265 nM) was added 30 minutes prior to TNF stimulation (50 ng/ml, Sigma). The cells were washed with ice cold PBS 15 minutes later then homogenized in lysis buffer (150 mM NaCl, 50 mM Tris, 0.5% Triton X-100, 3% sodium dodecyl sulfate, 1 mM EDTA, protease inhibitor cocktail, and phosphatase inhibitor cocktail I and II [Sigma]) using sonication. The samples were centrifuged at 14,000 rpm at 4 °C for 15 minutes and stored in -80 °C until analysis by western blotting.

### Spinal cord tissue collection and quantitative real-time PCR

Spinal cord tissue was harvested at 0, 2, 4, 8, 12, 18 and 24 hours after carrageenan injection. Rats were deeply anesthetized and spinal cords ejected from the vertebral column by hydroextrusion using a saline-filled syringe after decapitation. The ipsilateral half of the lumbar spinal cord (L3-L6) was dissected and rapidly frozen on dry ice and kept at −70 °C until analysis. The spinal cord samples were homogenized by sonication in TRIzol (Invitrogen, Carlsbad, CA, USA) and primary astrocytes were lysed by adding TRIzol to the wells. RNA was extracted from spinal cord and astrocyte samples as described previously [35]. The RNA was resuspended in Tris-EDTA buffer and the concentration measured using spectrophotometry (Nanodrop, Wilmington, DE, USA). The RNA was then reverse transcribed to complementary DNA (cDNA) and quantitative real-time PCR was performed with TaqMan Gene Expression Assays (Applied Biosystems, Foster City, CA, USA), both according to the manufacturer’s instructions, the relative mRNA levels were determined using the GeneAmp 7000 Sequence Detection system (Applied Biosystems). Predeveloped specific primers were used to detect rat and human FPR2/ALX (assay no. Rn03037051_gH and Hs02759175_s1), human GPR32 (assay no. Hs00265986_s1), and rat and human HPRT1 (assay no. Rn01527838_g1 and Hs01003270_g1; all from Applied Biosystems). Threshold cycle values in each sample were used to calculate the number of cell equivalents in the test samples using the standard curve method [35]. cDNA from TNF-stimulated astrocytes was used for FPR2/ALX standard curve. Data was normalized to HPRT1 expression and expressed either as relative expression units (REU) or as % of control values.

### Western blot analysis

Proteins in the cell culture samples were separated by NuPAGE 4-12% Bis-tris gel electrophoresis (Invitrogen, Carlsbad, CA, USA) and then transferred to nitrocellulose membranes (Invitrogen). After blocking nonspecific binding sites with 5% non-fat milk in 0.1% Tween 20/Tris buffered saline (TBS-T) for 1 hour at room temperature, the membranes were incubated with primary antibodies in TBS-T overnight at 4 °C (p-ERK cat. no 9106, total ERK cat. no. 9102, p-JNK cat. no. 9255, p-P38 cat. No 9216, total P38, cat. No 9212; all used at 1:1000, and β-actin, cat. no. 3700, 1:10,000, Cell Signaling, Danvers, MI, USA). Membranes were washed with TBS-T, blocked in 5% non-fat milk in and probed with secondary antibodies conjugated to horseradish peroxidase (1:7500, Cell Signaling) or near infrared fluorophores (1:20,000, Li-Cor Biosciences, Cambridge, UK) for 1 hour at room temperature. Immunopositive bands were detected using chemiluminescent reagents (Pico and Femto SuperSignal, Pierce, Rockford, IL, USA) and exposed to X-ray film or an Odyssey IR imaging system (Li-Cor Biosciences). Membranes were stripped between primary antibody incubations using Re-Blot Western blot recycling kit (Chemicon, Temecula, CA, USA). Densitometry analysis was done using Bio-Rad Quantity one software (Bio-Rad, Hercules, CA, USA) or Odyssey software v3.0.16 (Li-Cor Biosciences), by drawing a rectangle around each band and subtracting the background. Immunopositive bands were normalized relative to the bands for β-actin and the data was expressed as a percent change from control.

### Immunohistochemistry

Animals were deeply anesthetized with isoflurane and transcardially perfused with saline (200 ml) followed by freshly prepared 4% paraformaldehyde in 0.1 M PBS (pH 7.4; Sigma). Spinal cords from 3 naïve rats were removed, post-fixed in the same fixative for 4 hours and moved to PBS containing 20% sucrose for 24 hours and then 30% sucrose for 48 hours. The lumbar segments L4–L6 were dissected and transverse sections (30 µm) were cut with a cryostat and transferred to PBS. The free-floating sections were incubated with antibodies raised against the FPR2/ALX receptor (1:500, cat. no. NLS1878, Novus Biologicals, Littleton, CO, USA) in 0.5% Triton X-100 (Sigma) and 5% goat serum in PBS overnight at 4 °C. To confirm if FPR2/ALX was expressed in astrocytes, sections were double labeled with antibodies against an astrocyte marker (GFAP, 1:1,000, Chemicon). Binding sites were visualized with anti-mouse IgG antibody conjugated with Alexa-594 (1:250, cat. no. A11032, Invitrogen) and anti-rabbit IgG antibody conjugated with Alexa-488 (1:250, cat. no. A11058, Invitrogen). Images were captured using an Olympus fluorescence microscopy and overlay performed with Adobe Photoshop Creative suite (CS5; Adobe Systems Incorporated).

### Statistical analysis

All the data are presented as mean ± SEM and analyzed using GraphPad Prism 5 and 6 Software (La Jolla, CA, USA). Differences in mRNA levels and phosphorylation levels were assessed by one-way ANOVA followed by Bonferroni *post hoc* test. Behavioral data presented as hyperalgesic index, a derived value that defines the magnitude of inflammation-induced hypersensitivity (see above), were compared by one-way ANOVA followed by Bonferroni *post-hoc* test for multiple groups. Cytokine levels in CSF or cell culture media were compared for different treatments by one-way ANOVA followed by Fisher’s least significant difference (LSD) *post-hoc* test for comparing the groups of interest. The criterion for significance was set as p values < 0.05.

## Results

### Effect of i.t. injection of LXA4 and 17(R)-RvD1 on carrageenan-induced mechanical hypersensitivity and peripheral edema

**Figure 1:**
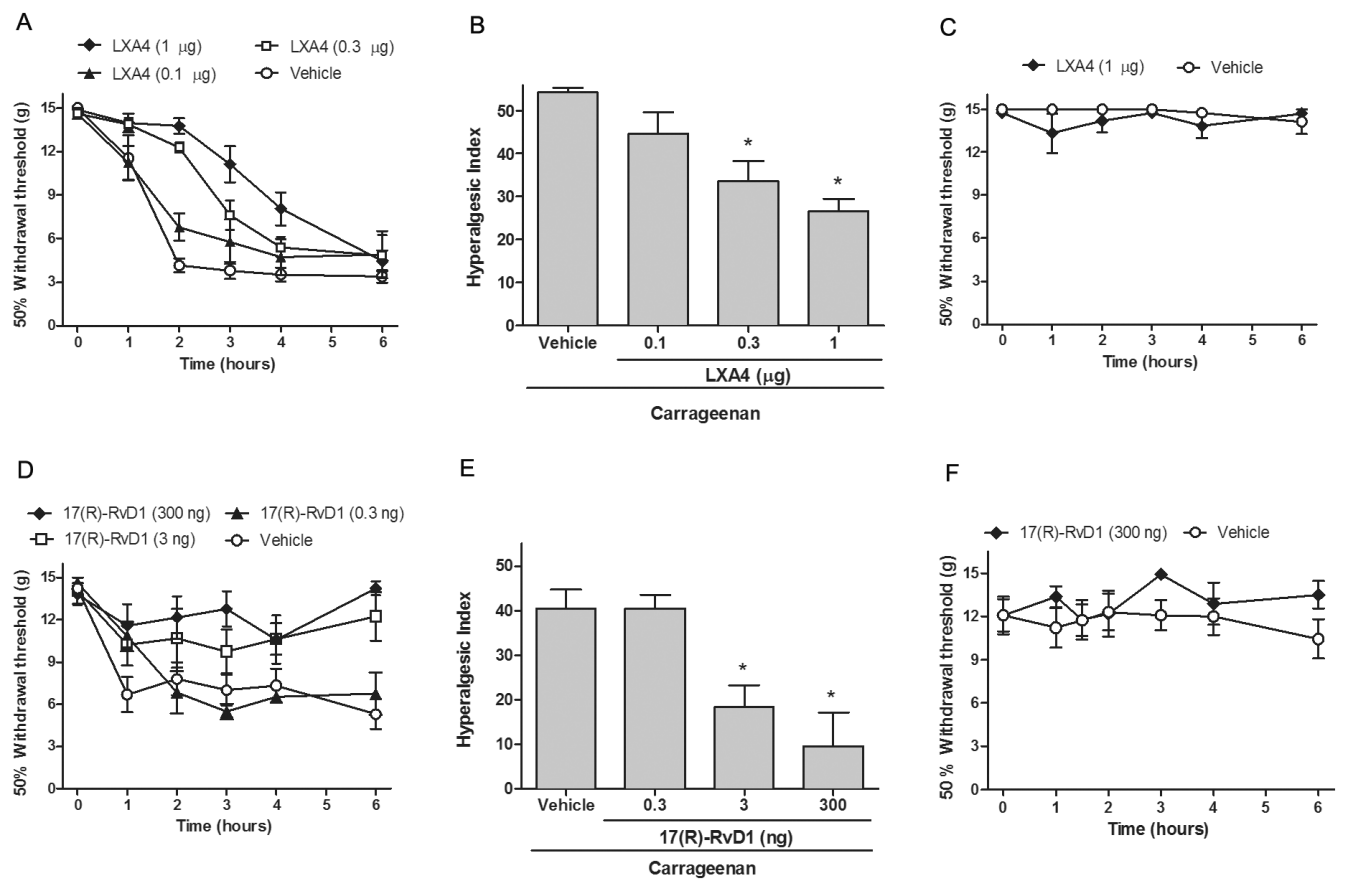
Intrathecal injection of LXA4 and 17(R)-RvD1 reduces carrageenan-induced mechanical hypersensitivity. Paw withdrawal thresholds plotted versus time are shown for the ipsilateral (A, D) and contralateral (C, F) hind paw following carrageenan administration. Intrathecal pretreatment with LXA4 (A) or 17(R)-RvD1 (D) reduced mechanical hypersensitivity as compared to vehicle, while i.t. injection of LXA4 (C) or 17(R)-RvD1 (F) had no effect on mechanical thresholds in the contralateral (non-inflamed) hind paw. The hyperalgesic index (see material and methods) calculated for 0–6 h was significantly reduced by LXA4 (B) and 17(R)-RvD1 (E) pretreatment. All data are presented as mean ± S.E.M, * represents a significant difference at p<0.05 as compared with i.t. vehicle.

Injection of carrageenan to the dorsal aspect of the paw results in a transient inflammation, apparent as an increase in paw volume, reddening of the skin and sensitivity to mechanical and thermal stimulation. In the current study, the carrageenan-induced inflammation appeared approximately 2 hours after injection and was resolved after 36 hours. Mechanical hypersensitivity was observed as early as 1 hour after the carrageenan injection (Figure 1A, D) and the tactile thresholds returned to baseline by the 24-hour time point (data not shown). Firstly, we examined if spinal injection of LXA4 or 17(R)-RvD1 attenuates inflammation-induced mechanical hypersensitivity and paw swelling. Lumbar i.t. injection of LXA4 (1 µg, n=9) 10 minutes prior to unilateral carrageenan-injection to the hind paw delayed the onset of carrageenan-induced mechanical hypersensitivity compared to vehicle-injected rats (Figure 1A). The lower LXA4 doses had only modest effects on mechanical hypersensitivity. Hyperalgesic index calculated for the time-period 0-6 hours was significantly lower in the groups that received LXA4 (0.3 µg, n=4 and 1 µg, n=9) compared to vehicle control (54±3, n=8) vs. (1 µg LXA4: 26±9 and 0.3 µg LXA4: 33±9, p<0.05) but not in the group that received 0.1 µg LXA4 intrathecally (n=3, p>0.05, Figure 1B). Unilateral carrageenan injection did not alter the tactile thresholds of the contralateral hind paw (Figure 1C and F) and i.t. LXA4 (1 µg) had no effect on the tactile thresholds of the contralateral hind paw (Figure 1C).

Lumbar i.t. injection of 17(R)-RvD1 (3-300 ng) 10 minutes prior to injection of carrageenan attenuated inflammation-induced mechanical hypersensitivity (Figure 1D, E). Intrathecal injection of 17(R)-RvD1 (3 ng: n=6, 300 ng: n=8) caused a significant decrease in carrageenan-induced hyperalgesic index (i.t. vehicle control: 41±4, n=12, vs. 3 ng 17(R)-RvD1: 18.5±5, and 300 ng 17(R)-RvD1: 10±7, p<0.05) (Figure 1E) while 0.3 ng 17(R)-RvD1 (n=4, p>0.05) did not. In addition, i.t. injection of vehicle (PBS) did not cause significant changes in tactile thresholds when compared to naive rats (data not shown). Intrathecal injection of vehicle or resolvins did not lead to changes in the tactile thresholds in the contralateral paw (Figure 1F).

**Figure 2:**
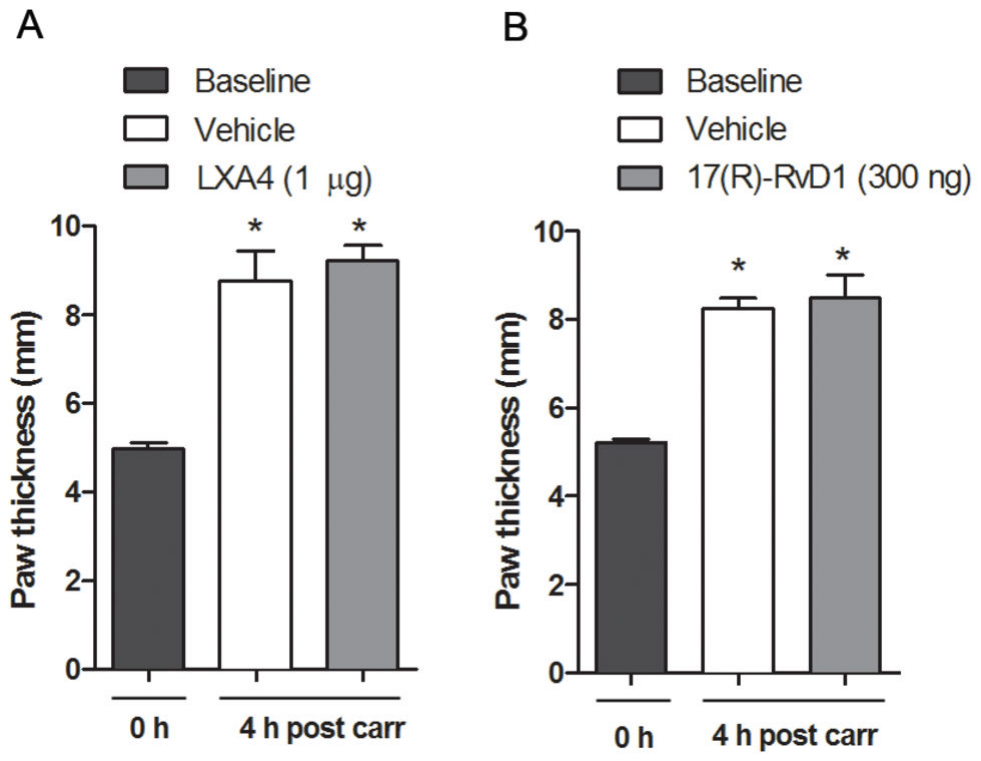
Intrathecal injection of LXA4 and 17(R)-RvD1 does not change carrageenan-induced inflammation. Bar graphs show that the increased paw size measured 4 h following carrageenan injection was not altered by pretreatment with LXA4 (A) or 17(R)-RvD1 (B) as compared to pretreatment with vehicle. All data are presented as mean ± S.E.M, * represents a significant difference at p<0.05 as compared with i.t. vehicle.

Paw swelling was assessed prior to and 4 hours after injection of carrageenan by measuring the thickness of the paw at the metatarsal level using vernier calipers. A significant increase in paw thickness was detected subsequent to injection of carrageenan (Figure 2A, B). However, i.t. injection of LXA4 (1 µg, Figure 2A) and 17(R)-RvD1 (300 ng, Figure 2B) did not reduce the paw thickness, indicating that lipoxin and resolvin receptors in the spinal cord are not involved in the regulation of peripheral edema.

### Effect of i.t. Injection of LXA4 and 17(R)-RvD1 on Carrageenan-Induced Spinal Cytokine Release

**Figure 3:**
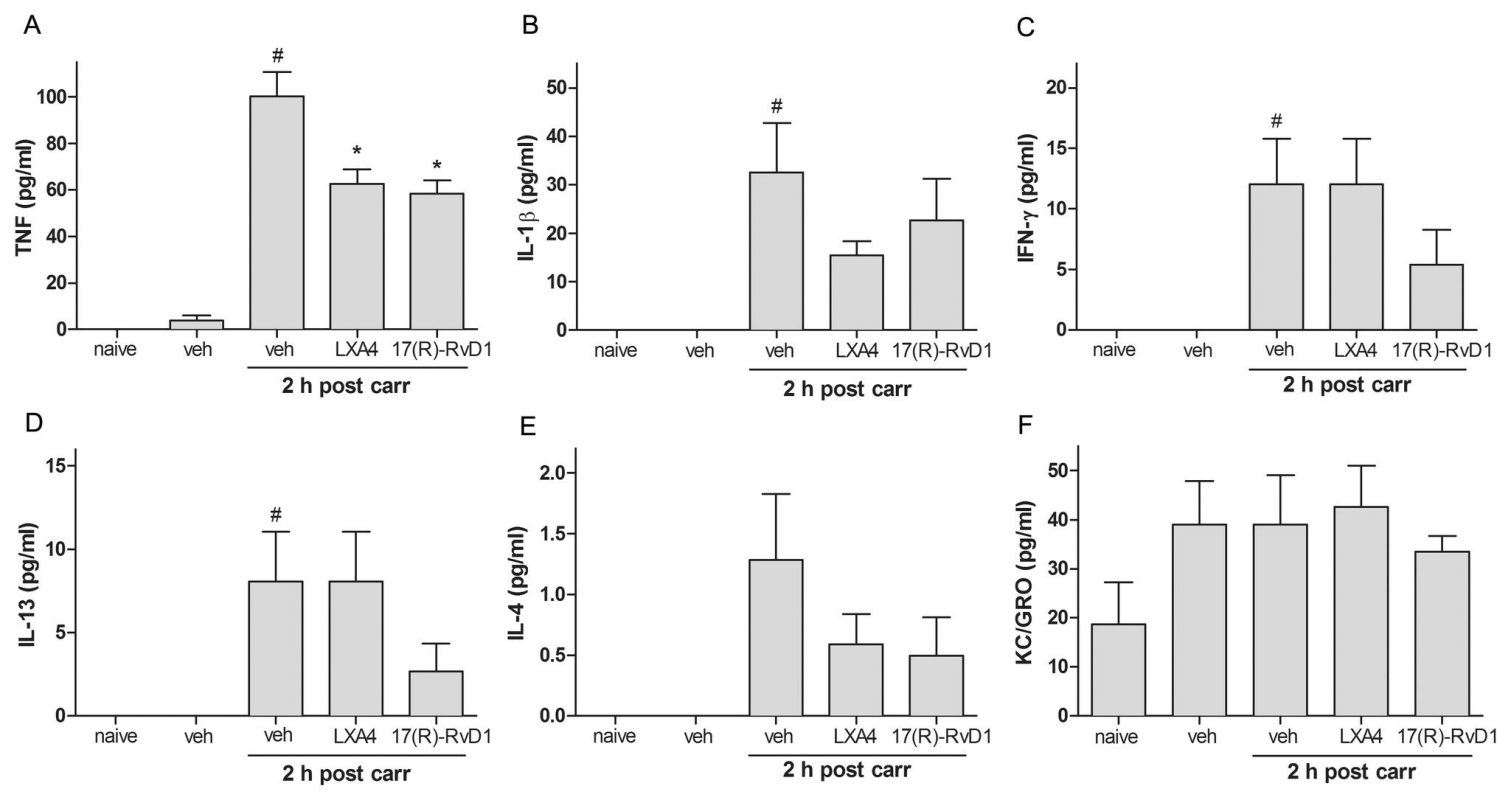
Intrathecal injection of LXA4 and 17(R)-RvD1 decrease carrageenan-induced TNF levels in the CSF. Bar graphs show the levels of cytokines in pg/ml in CSF from rats pretreated with i.t. RvD1 (300 ng) or LXA4 (1 µg) prior to carrageenan injection. (A) The carrageenan-induced increase in TNF levels was significantly reduced in rats pretreated with LXA4 and 17(R)-RvD1 (A). No significant effect of i.t. LXA4 or 17(R)-RvD1 was observed on CSF levels of IL-1β (B), IL-13 (C), IFN-γ (D), IL-4 (E), or KC/GRO (F). Each bar represents the mean ± S.E.M. ^#^ and * represent a significant difference at p<0.05 as compared to the i.t. vehicle (veh) treated group before and 2 hours post carrageenan, respectively.

We next examined if i.t. administration of LXA4 or 17(R)-RvD1 altered the release of TNF, IL-1β, IL-4, IL-13, KC/GRO and IFN-γ following peripheral carrageenan injection. CSF was collected by lumbar puncture. A pilot study, in which CSF was collected at different time-points over a 24-hour period, revealed that TNF, IL-1β, IL-13, and IFN-γ were elevated in CSF 2 and 4 hours after carrageenan-injection (data not shown). Based on this LXA4 (1 µg) and 17(R)-RvD1 (300 ng) were injected intrathecally 10 minutes before injection of carrageenan to the paw, and CSF was withdrawn by lumbar puncture 2 hours after carrageenan injection. While no significant change was found in KC/GRO and IL-4 levels subsequent to carrageenan injection (Figure 3E, F), an increase in TNF, IL-1β, IL-13, and IFN-γ concentrations were detected in CSF 2 hours after injection of carrageenan to the hind paw as compared to the vehicle (PBS) injected control group (Figure 3A-D). Pretreatment with i.t. LXA4 and 17(R)-RvD1 significantly attenuated carrageenan-induced spinal TNF release (PBS: 100.4±10.4, n=6, vs. LXA4: 62.7±6.2 pg/ml, n=7, p<0.05, 17(R)-RvD1: 58.4±5.7 pg/ml, n=6, p<0.05, Figure 3A). At the 2-hour time point after carrageenan injection, no significant effect of i.t. LXA4 and 17(R)-RvD1 was observed on CSF levels of IL-1β, IL-4, KC/GRO, IL-13 and IFN-γ (p>0.05, Figure 3B-F).

### Effect of LXA4 and 17(R)-RvD1 on LPS and IFN-γ-induced TNF release in rat astrocyte culture

**Figure 4:**
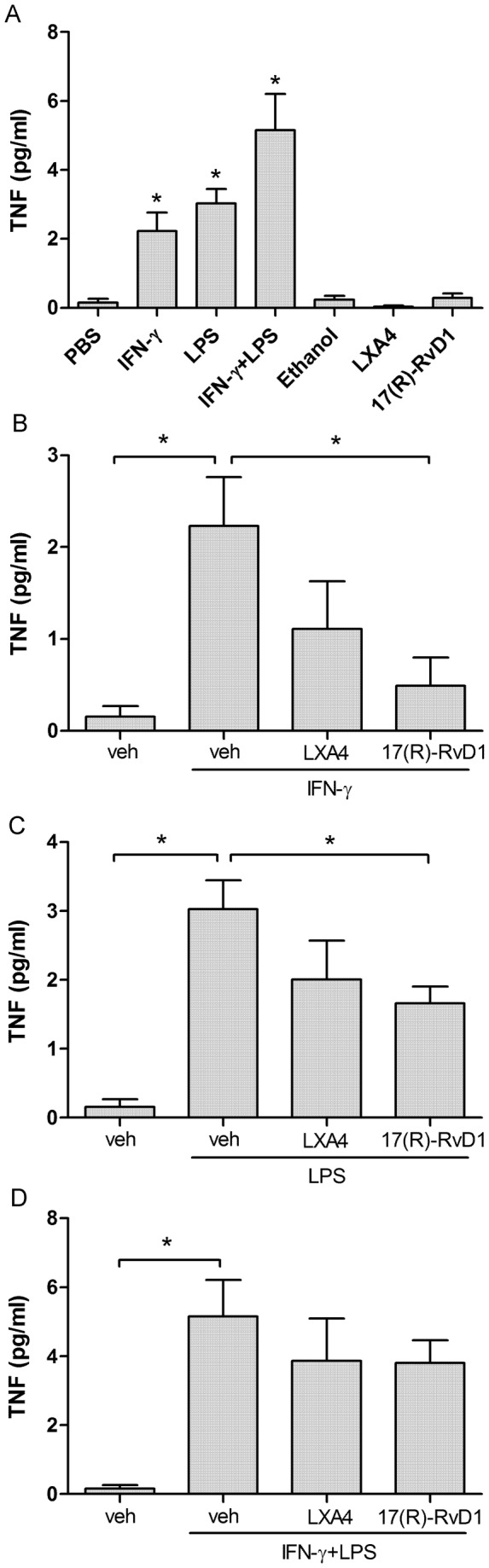
17 (R)-RvD1 attenuate IFN-γ and LPS induced TNF induction in rat primary astrocytes. Bar graphs showing levels of TNF in media from primary rat astrocyte cultures (A) following stimulation with IFN-γ (1000 U/ml, 24 h), LPS (2 µg/ml, 24 h) or the combined effect of IFN-γ (1000 U/ml, 4 h) followed by LPS (2 µg/ml, 20 h). Pretreatment with 17(R)-RvD1 (500 nM, 30 min) significantly reduced IFN-γ (B) and LPS-induced (C)-TNF release. No significant effect was seen after pretreatment with LXA4 (500 nM, 30 min). Each bar represents mean ± S.E.M, * represents a significant difference at p<0.05 as compared to PBS (A) or indicated in the figure (B-D).

In rat primary spinal astrocyte cultures, LPS (2 µg/ml) and IFN-γ (1000 U/ml) stimulation alone (24 hours), as well as IFN-γ (1000 U/ml) priming (4 hours) prior to LPS (2 µg/ml, 20 hours) stimulation induced TNF release compared to control conditions (control PBS: 0.16±0.11 pg/ml vs. IFN-γ: 2.3±0.5 pg/ml, LPS: 3±0.4 pg/ml, and LPS+IFN-γ: 5.16±1 pg/ml, p<0.05) (Figure 4A). Pretreatment with 17(R)-RvD1 (500 nM, 30 minutes) decreased both IFN-γ-induced TNF release (0.5±0.3 pg/ml, p<0.05, Figure 4B) and LPS-induced release of TNF (1.67±0.2 pg/ml, p<0.05, Figure 4C). However, 17(R)-RvD1 did not prevent TNF release in cells primed with IFN- γ prior to stimulation with LPS (Figure 4C). Pretreatment with LXA4 (500 nM, 30 minutes), on the other hand, had no significant effect on TNF release in response to any of the stimuli (Figure 4B, C, D). Addition of LXA4 and 17(R)-RvD1, PBS or 0.22% ethanol (vehicle for LXA4 and 17(R)-RvD1) did not alter TNF levels in the cell media from unstimulated cells (Figure 4A).

### FPR2/ALX expression in the rat spinal cord and cultured rat and human spinal astrocytes

**Figure 5:**
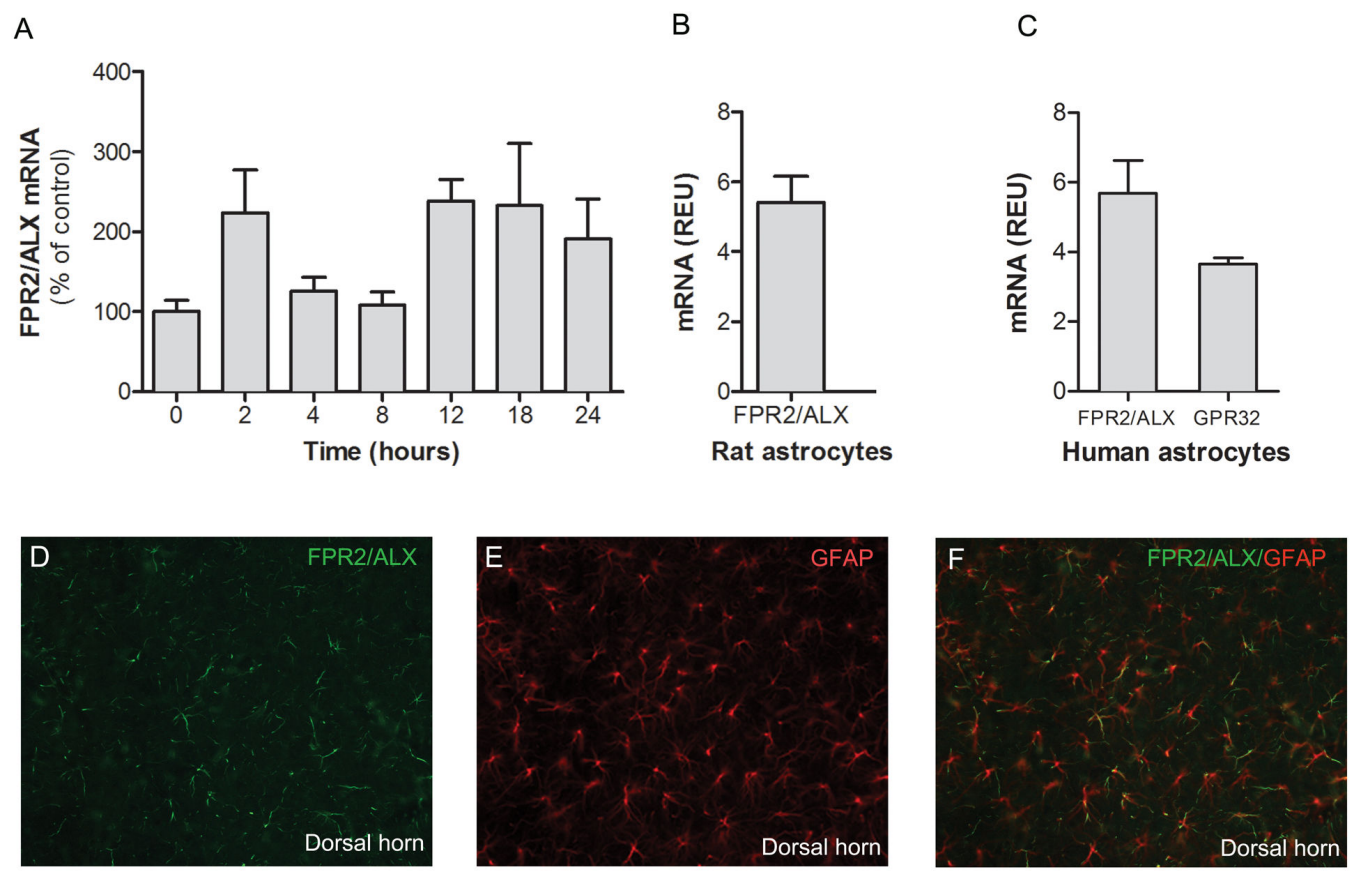
FPR2/ALX mRNA is present in the rat spinal cord and cultured spinal astrocytes. Bar graphs showing the expression of (A) FPR2/ALX mRNA in the rat ipsilateral spinal cord plotted versus time following carrageenan injection to the hind paw, presented as a percent of mRNA levels in control (naïve) rat spinal cord. Each bar represents the mean ± S.E.M, n= 6-10. * represents a significant difference at p<0.05 as compared with naïve spinal cord. FPR2/ALX mRNA is expressed in the rat (B) and human astrocytes (C). GPR32 mRNA is expressed in human astrocytes (C). mRNA levels are expressed as relative units and each bar represents the mean ± S.E.M for three repeats. The immunohistochemical images show the expression of FPR2/ALX (D), the astrocyte marker GFAP (E) and the colocalization of FPR2/ALX and GFAP (F) in naïve rat lumbar spinal cord.

In order to examine if the expression of the 17(R)-RvD1 and LXA4 receptor FPR2/ALXR is altered in the spinal cord subsequent to peripheral inflammation, we assessed spinal FPR2/ALXR mRNA levels in naïve spinal cords and at different time points after carrageenan-induced inflammation. FPR2/ALX mRNA was readily detected in naïve spinal cord and the levels did not change significantly following carrageenan-injection to the paw (Figure 5A). Immunohistochemistry showed that FPR2/ALX is expressed in the naïve rat spinal cord and that FPR2/ALX immunoreactivity colocalizes with the astrocyte marker GFAP (Figure 5D-E).

In order to determine if primary cultures of astrocytes express FPR2/ALX, mRNA from both rat and human primary astrocyte cultures were isolated. Quantitative real time PCR showed that rat and human primary astrocytes expressed FPR2/ALX mRNA (Figure 5B). In addition, human astrocytes expressed GPR32 mRNA (Figure 5C)

### 17 (R)-RvD1 effect on TNF-induced MAPK activation in astrocyte culture

**Figure 6:**
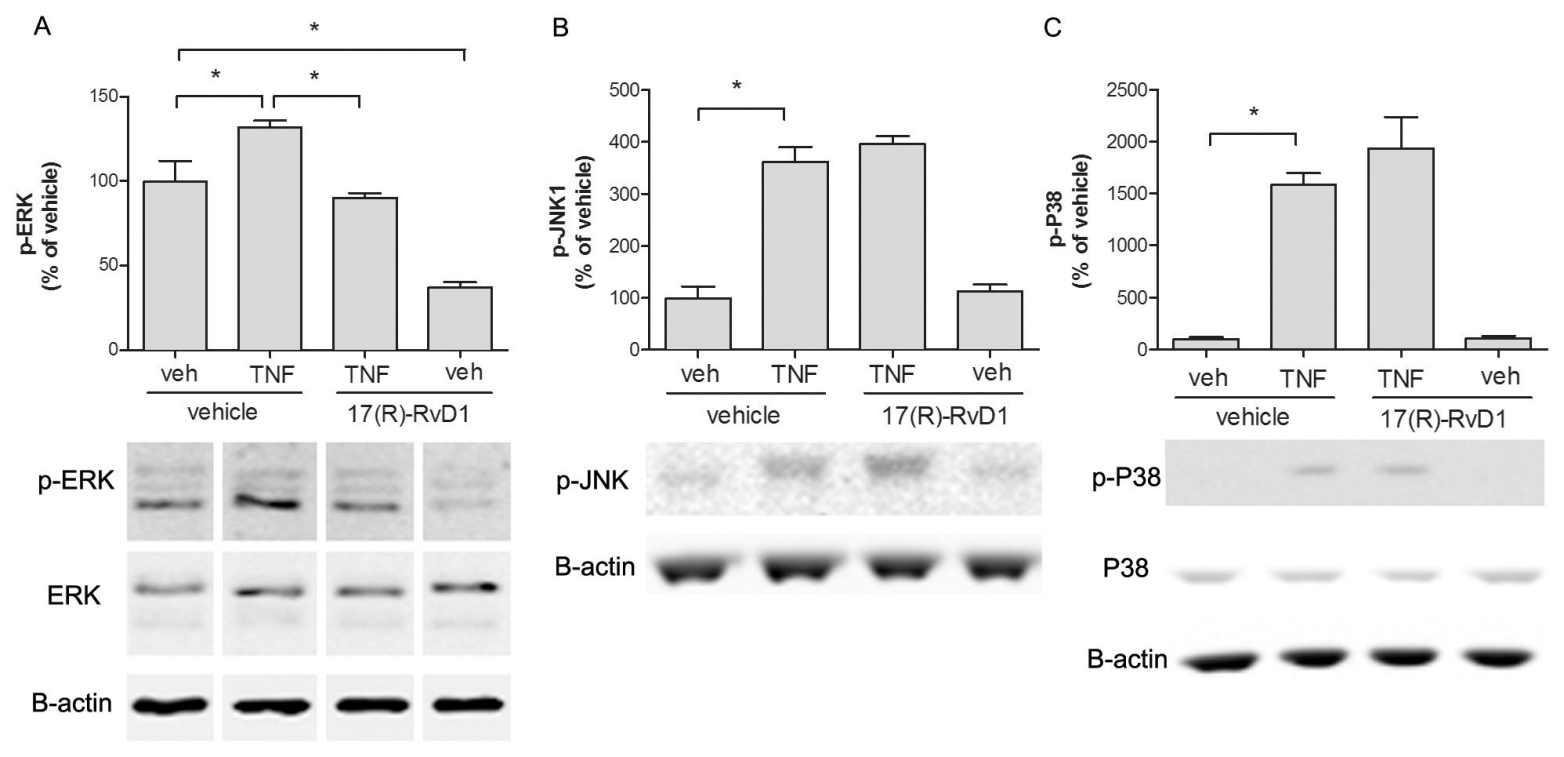
17(R)-RvD1 reduced TNF-induced ERK activation in human primary astrocytes. Bar graphs and representative western blots showing MAPK phosphorylation levels in control and TNF (50 ng/ml) stimulated cells. In astrocytes, 17(R)-RvD1 (265 nM) inhibited TNF-induced ERK (A), but not p38 (B) or JNK (C) phosphorylation. Each bar represents the mean ± S.E.M for three repeats. * represents p<0.05 for comparisons indicated in the figure.

We next assessed if 17(R)-RvD1 attenuates TNF-induced MAPK activation in human spinal astrocytes culture. After 24 hours serum starvation the astrocytes were treated with 17(R)-RvD1 (265 nM) 30 minutes prior to TNF (50 ng/ml) stimulation for 15 minutes. TNF stimulation induced a statistically significant increase in phosphorylation of ERK1/2, JNK1 and p38 in human primary astrocytes (Figure 6A-C). TNF-induced JNK1 and p38 activation was unaltered in the presence of 17(R)-RvD1 (Figure 6B, C). However, 17(R)-RvD1 not only reduced TNF-induced ERK1/2 activation (132±3.79 vs. 90±2.89%, p<0.05, n=3) but also reduced basal ERK1/2 phosphorylation (99.7±12.3 vs. 37.3±2.85%, p<0.05, n=3, Figure 6A).

## Discussion

The present study demonstrates that injection of LXA4 and 17(R)-RvD1 into the CSF reduces inflammation-induced mechanical hypersensitivity. Intrathecal injection of these agents had no effect on the peripheral inflammation, thus our data suggests that lipoxins and resolvins are anti-nociceptive via spinal mechanisms, and that they can attenuate hypersensitivity even in the presence of an ongoing peripheral inflammation. Furthermore, carrageenan-induced inflammation leads to spinal release of TNF, which was prevented by i.t. pretreatment with LXA4 and 17(R)-RvD1. Spinal astrocytes express FPR2/ALX *in vivo* and in culture, and we found that 17(R)-RvD1 attenuated stimulus-induced TNF release in astrocyte culture. Hence, it is plausible that LXA4 and 17(R)-RvD1, which both have been identified as FPR2/ALX ligands, play important roles in the regulation of spinal sensitization and pain processing at least partly through interference with cytokine release.

In the current study, we found that spinal delivery of LXA4 delays the onset of carrageenan-induced mechanical hypersensitivity in a dose-dependent manner. This finding adds to our previous work, which revealed that i.t. LXA4, LXB4 and aspirin-triggered LXA4 (15-epi-lipoxin A4) delay the onset of thermal hyperalgesia induced by injection of carrageenan to the hind paw [2]. Previous work has shown that 17(R)-RvD1 reduces CFA and capsaicin-induced hyperalgesia in the rat when delivered systemically or intradermally [10,11]. However, the anti-nociceptive effect of 17(R)-RvD1 in the spinal cord has not previously been examined and here we show that pretreatment with i.t. 17(R)-RvD1 prevents inflammation-induced mechanical hypersensitivity in a dose-dependent manner. Compared to LXA4, the anti-nociceptive action of 17(R)-RvD1 lasted longer and completely prevented the induction of carrageenan-mediated hypersensitivity within the 6-hour test window. In our previous work, we found that the aspirin-triggered form of LXA4 induced a longer-lasting anti-hyperalgesic effect compared to native LXA4 [2]. Though we cannot make direct comparison between LXA4 and 17(R)-RvD1, as these are different lipid species, it is possible that the longer-lasting anti-nociceptive effect of 17(R)-RvD1 is a matter of stability. We chose to study 17(R)-RvD1 as it resists hydrolysis and thus is more stable than the native 17(S)-RvD1 form (commonly referred to as RvD1). Of note, 17(S)-RvD1 also has spinal anti-nociceptive effects as evidenced by studies showing that i.t. RvD1 attenuate hypersensitivity subsequent to surgery in rats [15] and CFA-induced peripheral inflammation in mice [4,9]. Thus there is accumulating evidence for a role of LXA4 and RvD1 in pain transmission and our findings that i.t. LXA4 and 17(R)-RvD1 potently attenuate inflammation-induced mechanical hypersensitivity in rats further suggests a role for these anti-inflammatory lipid mediators in interfering with spinal pain processing.

Given the anti-inflammatory properties of lipoxins and resolvins [8,25,36], it is not surprising that local or systemic injection of these factors prior to carrageenan injection reduces paw edema [2,4,37]. One could speculate that the anti-nociceptive effect observed in the current study was due to effects outside the spinal cord. However as i.t. injection of these mediators did not alter paw edema it is unlikely that the anti-nociceptive effect was due to systemic redistribution. Thus lipoxins and resolvins modulate nociception, not only at the site of inflammation, but also by regulating pain processing in the central nervous system, at a site remote from the ongoing inflammatory reaction. Of note, while the spinal cord is the most likely site of action after i.t. delivery, we cannot rule out that the anti-nociceptive effect was also mediated subsequent to diffusion of the drugs to the DRG or supraspinal sites.

The ability of LXA4 and 17(R)-RvD1 to attenuate mechanical hypersensitivity at the level of the spinal cord may depend on several mechanisms. Since these classes of anti-inflammatory lipid mediators reduce cytokine production in peripheral immune cells [38,39], it is possible that they display the same action on spinal glia cells. An increasing number of reports indicate that cytokines such as TNF and IL-1β are important factors in spinal pain processing in models of inflammation and nerve injury-induced pain [40]. Both TNF and IL-1β mRNA and protein expression are increased in the spinal cord following induction of peripheral inflammation, and blocking the spinal action of these cytokines attenuates inflammation-induced pain [41,42]. In the current work, we found that injection of carrageenan to the hind paw caused a rapid increase of TNF, IFN-γ, IL-1β and IL-13 levels in CSF. Importantly, i.t. injection of LXA4 and 17(R)-RvD1 partially prevented carrageenan-induced spinal TNF-release, pointing to one potential mechanism by which these factors may reduce hypersensitivity. In agreement with our finding, both LXA4 and 17(R)-RvD1 have been shown to attenuate cytokine production in other experimental models of pain. For example, spinal LXA4 and its aspirin-triggered form reduced TNF, IL-1β and IL-6 mRNA and protein levels in the DRG in a neuropathic pain model [12] and in the spinal cord in a model of bone cancer-induced pain [13]. LXA4 was also shown to attenuate zymosan-induced TNF production in joints following intraarticular injection [43]. Moreover, systemic post-treatment of 17(R)-RvD1 reduced the levels of TNF and IL-1β in the rat hind paw in the CFA model [11].

Astrocytes play an important role in the development and maintenance of pathological pain by contribution to spinal sensitization [44,45] and accordingly astrocyte inhibitors attenuate hypersensitivity in pain models [46,47]. Thus biochemical processes in astrocytes represent novel targets for pain therapeutics. Upon stimulation with different agents, e.g. LPS and certain cytokines, astrocytes in culture respond by producing cytokines such as IL-6 and TNF [48-51]. LPS activates the toll like receptor 4 (TLR4) [52], which is expressed on astrocytes and microglia in the CNS [53]. In agreement with previous *in vitro* and *in vivo* work showing that both 17(S)-RvD1 and 17(R)-RvD1 attenuates cytokine production [8,54], we found that TNF release induced by IFN-γ and LPS, but not the two combined, in astrocytes was attenuated in the presence of 17(R)-RvD1. It is possible that a higher 17(R)-RvD1 concentration is required to block TNF release in the latter case, or that the mechanisms of release are altered in the presence of both LPS and IFN-γ. In previous work, LXA4 and AT-LXA4 attenuated LPS-induced TNF release in microglia cultures [55]. However, although 17(R)-RvD1 prevented TNF release in our study, LXA4 did not. This could be due to differences in stability or potency. In the current *in vitro* experiment the same concentration of LXA4 and 17(R)-RvD1 was used. However, in the *in vivo* studies a higher dose LXA4 was required to generate an anti-nociceptive effect similar to what was achieved by 17(R)-RvD1. Thus it is possible that a higher concentration of LXA4 is needed also in *in vitro* studies for blockage of TNF release.

It is commonly reported that primary astrocyte cultures contain up to 5% microglia. Thus, the source and magnitude of TNF release may not only be attributed to astrocytes in such cultures as microglia have higher capacity to produce TNF in response to LPS stimulation [56]. Interestingly, several studies report failure to evoke TNF production in pure astrocytes cultures [57,58]. We have recently developed a modified protocol for establishment of primary rat spinal astrocytes in which microglia contamination is significantly reduced [34]. In our nearly microglia-free cultures, LPS and IFN-γ induced only a modest release of TNF. However, priming the cells with IFN-γ for 4 hours before addition of LPS, which has been shown to increase TLR4 gene expression [59], resulted in a more pronounced LPS-induced TNF release, which is in agreement with previous work [49,60]. Thus it is possible that the magnitude of astrocytes’ response to LPS stimulation is highly condition dependent. Interestingly, increased levels of IFN-γ were noted in CSF following peripheral inflammation and thus it is possible that IFN-γ drives upregulation of TLR4 on spinal astrocytes enabling endogenous TLR4 ligands to drive spinal TNF release from these cells, or that IFN-γ directly mediates TNF-release in the spinal cord. As we noted only a modest TNF release from the cultured astrocytes in the current work, another possibility is that microglia is the main source of TNF *in-vivo, and* that LXA4 and 17(R)-RvD1 reduce TNF release through a direct or an indirect effect on microglia.

FPR2/ALX has a wide expression on cells of immune and non-immune origin including monocytes, macrophages, synovial fibroblasts and epithelial cells in different species [16]. Less is known about GPR32, which is a human orphan receptor [61] that has not been identified in rodents. Higher levels of FPR2/ALX mRNA and LXA4 have been reported in the synovium and synovial fluid, respectively, of rheumatoid arthritis patients compared to osteoarthritis patients, suggesting a stronger involvement of the ligand-receptor system in inflammatory conditions [62]. We detected FPR2/ALX mRNA in naïve rat spinal cord, but its expression did not change during the 24-hour course of carrageenan-induced inflammation. The relatively short duration and moderate degree of inflammation in the carrageenan model may not be sufficient to drive upregulation of FPR2/ALX mRNA in the spinal cord, however, the effect of these lipid mediators is clearly not dependent on increased receptor expression. Immunohistochemical analysis of the spinal cord showed FPR2/ALX protein expression on astrocytes in the dorsal horn of spinal cord of naïve rats, which is in line with previous reports [2]. Further, FPR2/ALX mRNA was detected in cultured human and rat primary astrocytes indicating that this location is conserved between those species, at least in primary cells. FPR2/ALX expression has also been identified in human astrocytoma cell lines [20,63]. Moreover, we detected GPR32 mRNA in human primary spinal astrocyte cultures providing the possibility that LXA4 and 17(R)-RvD1 can be acting through two receptor systems on the same cell type in humans. The expression of both GPR32 and FPR2/ALX in human astrocytes makes this cell culture a valuable tool for studies aimed at investigating mechanisms of action of LXA4 and 17(R)-RvD1.

The MAPK family members ERK, JNK and p38 are activated in the spinal cord in a time and cell dependent manner in many experimental models of pain. In astrocytes, particularly ERK and JNK activation has been associated with spinal sensitization [31]. Interestingly, ERK and JNK are activated in response to cytokines [64,65] and their activation, in turn, has been implicated in further cytokine release [66,67]. Hence we asked if 17(R)-RvD1, in addition to attenuating LPS-induced TNF release, can suppress cytokine-induced MAPK activation. Using human primary astrocytes, we found that TNF-stimulation activated ERK, JNK and p38, of which ERK activation was the strongest. 17(R)-RvD1 attenuated TNF-evoked ERK but not p38 or JNK-activation. Actions of resolvins of the E and D series have previously been coupled to inhibition of ERK activation. RvD1 and 17(R)-RvD1 block histamine-induced ERK activation in rat conjunctival goblet cells [68] and the anti-nociceptive action of RvE1 has been coupled to suppression of ERK activation in DRG [4]. Since ERK activation in astrocytes has been linked to augmentation of central sensitization in pain conditions our findings suggest the possibility that 17(R)-RvD1 interference with ERK activation dampens central sensitization.

Our data, taken together, provides further support for a modulatory role of lipoxins and resolvins in spinal nociceptive processing. These lipid metabolites exert potent anti-nociceptive actions by interference with intracellular signaling pathways involved in pain processing and suppression of TNF release in the spinal cord in response to peripheral inflammation. Furthermore, the expression of their receptors in the spinal cord, specifically on astrocytes, points to a possible role these endogenous ligands can play in pain conditions. Most importantly, lipoxins and resolvins appear to have multiple sites of action, putting them in an intriguing position in the search for novel pain therapeutics.
